# Evaluating the safety of two human experimental intestinal ischemia reperfusion models: A retrospective observational study

**DOI:** 10.1371/journal.pone.0253506

**Published:** 2021-06-18

**Authors:** Inca H. R. Hundscheid, Dirk H. S. M. Schellekens, Joep Grootjans, Marcel Den Dulk, Ronald M. Van Dam, Geerard L. Beets, Wim A. Buurman, Kaatje Lenaerts, Joep P. M. Derikx, Cornelis H. C. Dejong

**Affiliations:** 1 Department of Pathology, Maastricht University Medical Centre+, Maastricht, The Netherlands; 2 NUTRIM School for Nutrition, Toxicology and Metabolism, Maastricht University Medical Centre+, Maastricht, The Netherlands; 3 Department of Surgery, Maastricht University Medical Centre+, Maastricht, The Netherlands; 4 Department of Gastroenterology and Hepatology, Amsterdam University Medical Centre, University of Amsterdam, Amsterdam, the Netherlands; 5 Department of Surgery, RWTH University Hospital Aachen, Aachen, Germany; 6 Department of Surgery, The Antoni van Leeuwenhoek Netherlands Cancer Institute, Amsterdam, The Netherlands; 7 GROW School for Oncology and Developmental Biology, Maastricht University Medical Centre+, Maastricht, The Netherlands; 8 MHeNs School for Mental Healthy and Neuroscience, Maastricht University Medical Centre+, Maastricht, The Netherlands; 9 Department of Pediatric Surgery, Emma Children’s Hospital, Amsterdam University Medical Centre, University of Amsterdam, Free University of Amsterdam, Amsterdam, The Netherlands; Ohio State University Wexner Medical Center Department of Surgery, UNITED STATES

## Abstract

**Background:**

We developed a jejunal and colonic experimental human ischemia-reperfusion (IR) model to study pathophysiological intestinal IR mechanisms and potential new intestinal ischemia biomarkers. Our objective was to evaluate the safety of these IR models by comparing patients undergoing surgery with and without in vivo intestinal IR.

**Methods:**

A retrospective study was performed comparing complication rates and severity, based on the Clavien-Dindo classification system, in patients undergoing pancreatoduodenectomy with (n = 10) and without (n = 20 matched controls) jejunal IR or colorectal surgery with (n = 10) and without (n = 20 matched controls) colon IR. Secondary outcome parameters were operative time, blood loss, 90-day mortality and length of hospital stay.

**Results:**

Following pancreatic surgery, 63% of the patients experienced one or more postoperative complications. There was no significant difference in incidence or severity of complications between patients undergoing pancreatic surgery with (70%) or without (60%, *P* = 0.7) jejunal IR. Following colorectal surgery, 60% of the patients experienced one or more postoperative complication. Complication rate and severity were similar in patients with (50%) and without (65%, *P* = 0.46) colonic IR. Operative time, amount of blood loss, postoperative C-reactive protein, length of hospital stay or mortality were equal in both intervention and control groups for jejunal and colon IR.

**Conclusion:**

This study showed that human experimental intestinal IR models are safe in patients undergoing pancreatic or colorectal surgery.

## Introduction

Intestinal ischemia reperfusion (IR) is a frequent occurring phenomenon following multiple clinical situations. It is the result of thromboembolic occlusion of the mesenteric arterial blood supply causing acute mesenteric ischemia, followed by restoration of the blood flow (reperfusion). More often intestinal IR is observed as part of intestinal hypoperfusion in patients suffering from shock, trauma, sepsis or major surgery [1–4]. These severe stress situations lead to a significant reduction of mesenteric blood flow which preserves central hemodynamic stability, but results frequently in nonocclusive mesenteric ischemia. Paradoxically, the subsequent return of oxygenated blood during reperfusion aggravates ischemia induced tissue damage [3,5]. Intestinal IR is characterized by a decrease of gut barrier function. Dysfunction of the gut barrier has been implicated as a major contributor to the systemic inflammatory responses following intestinal IR, which can evolve to multiple organ failure and death [6,7]. These systemic inflammatory responses account for the high morbidity and mortality rates of 60–80% associated with intestinal IR [1,8].

It is intriguing that the morbidity and mortality rates of intestinal IR did not improve over the last decades, despite improvements in surgery, interventional radiology and intensive care medicine [8]. This is partly due to the lack of insight in the pathophysiological processes during intestinal IR and consequently the shortcoming in preventive and/or therapeutic options. Next, these grave outcomes are closely linked to a delay in diagnosis. This is mainly attributable to the nonspecific clinical presentation in combination with a paucity of early, non-invasive diagnostic markers for intestinal ischemia [9–11]. Animal models have been indispensable to obtain more insight into the mechanisms of intestinal IR. However, due to differences between various animals and models most results cannot be translated to the clinical setting [12]. For this purpose our group developed in vivo models for intestinal IR in which assessment of IR injury was performed in humans, allowing for a more direct translation of the results to patients [13,14].

In our experience, the models seemed safe and harmless for the individual participating patients with no serious adverse events reported. However, objective evidence is needed regarding the long-term post-operative outcomes and complication rates of these patients as a group, compared to a group of control patients undergoing the same type of surgery, performed by the same surgeons in the same hospital without being exposed to IR according to the above mentioned IR-models.

This study aimed at evaluating the safety of the human intestinal IR models by comparing the post-operative outcomes and complication rates between the IR-groups and a group of matched control patients.

## Materials and methods

### Ethics

The study was approved by the Medical Ethical Committee of Maastricht University Medical Center and was conducted according to the revised version of the Declaration of Helsinki (October 2013, Fortaleza). Written informed consent of all patients participating in the IR studies was obtained. The responsible ethics committee granted an exemption from requiring informed consent for the retrospective gathering and analysis of medical data from control patients, who did not participate in the IR studies.

### Experimental IR procedures and control patients

The studies were carried out in the Maastricht University Medical Center (MUMC) in Maastricht, the Netherlands from 2008 until completion of data analysis in 2015. This is a high volume, tertiary referral center for hepato-pancreato-biliary (HPB) and colorectal surgery with 45–65 pancreatic resections per year and 90–100 colorectal procedures.

#### Jejunal IR

To investigate human small intestinal IR, patients undergoing a pancreatoduodenectomy for benign or malignant pancreatic head tumors were included at the Maastricht University Medical Center from December 2008 until August 2010. In this time period 83 pancreatic resections were performed. From this cohort of patients, ten patients were included in the jejunal ischemia-reperfusion group. Six centimeter of healthy small intestine from these patients was exposed to 60 minutes of ischemia (60I), followed by 30 and 120 minutes of reperfusion according to the human jejunal IR model as described elsewhere [13,15–17].

In summary, in patients undergoing a pancreatoduodenectomy, a six cm piece of healthy small intestine, usually resected in continuity with the (oncologic) specimen, was isolated from the remaining bowel and mesentery. This was achieved by staple transection of the intestine and clamping and ligation of the collateral mesenterial vessels, leaving only a small vascular stalk with one mesenteric supplying arteriole and draining venule. Acute, complete ischemia of this isolated intestinal segment was achieved by placing two atraumatic vascular clamps across this vascular stalk, for a time period of 60 minutes. Ischemia was macroscopically confirmed by blue discoloration of the intestine and absence of peristalsis. After the period of ischemia, the first sampling of intestinal tissue took place by resecting one third of the isolated ischemic jejunum, using a linear cutting stapler (GIA; Medtronic, Eindhoven, the Netherlands). After removing the clamps and visual conformation of adequate reperfusion, subsequent segments (two cm each) of the reperfused isolated jejunum were resected similarly after 30 minutes of reperfusion (30R) and 120 minutes of reperfusion (120R). Internal control tissue was obtained by resecting two cm of jejunum, which remained untreated during surgery [13,16]. This segment underwent similar surgical handling as the isolated part of the jejunum but was not exposed to IR. All tissue samples were immediately formalin-fixed for immunohistochemical analysis. Arterial blood drawn from the radial artery, and venous blood drawn from the venule draining the isolated jejunal segment, was sampled before ischemia, immediately after ischemia and at 30R and 120R, to assess concentration gradients across the isolated jejunal segment [15].

#### Colon IR

For the experimental colon IR protocol, patients undergoing low anterior resection or abdominoperineal resection for colorectal cancer were included from March 2009 until October 2010 at the Maastricht University Medical Center. In this time period 153 colorectal operations were performed. From this cohort of patients, ten patients were included in the colonic ischemia-reperfusion group. In these patients, a six cm piece of healthy colon was subjected to 60 minutes of ischemia (60I), followed by 30 and 60 minutes of reperfusion according to the human colon IR model previously described [16,18].

The colon IR protocol was executed analogue to the small intestinal IR protocol. The isolated colonic segment was exposed to 60 minutes of ischemia, followed by 30 minutes (60I30R, short reperfusion) and 60 minutes of reperfusion (60I60R, prolonged reperfusion). At the end of the IR protocol, internal colonic control tissue was obtained by resecting a small part of the large intestine just proximal from the site of initial transection, not exposed to IR [16,18]. Because of differences in duration of surgery, only 60R in the colonic IR model could be achieved, as opposed to 120R in the small intestinal IR model.

During the IR protocol in both experimental models, surgery proceeded in accordance with the respective standard operating procedures for pancreatoduodenectomy, low anterior resection or abdominoperineal resection. For both the jejunal IR and the colon IR study, patients who refused or were unable to provide informed consent were excluded. Furthermore patients with other underlying intestinal diseases were excluded as well [13,16]. Other comorbidities, such as systemic vascular diseases or smoking were not used to exclude patients in order to obtain a study population which resembles daily surgical practice the most.

#### Control patients

To evaluate the safety of the IR models, we extracted data from the medical charts of patients, who underwent the same type of surgery in the same hospital and time period as the IR-patients, but were not exposed to the IR protocols. For every patient subjected to the IR-model, two control patients were selected. These patients were matched for age, sex, tumor characteristics/classification, operation period and Charlson comorbidity index.

### Data collection and definitions of outcome

Health record files of all the patients (study group and control group) were studied to obtain clinical parameters regarding sex, age, diagnosis, medical history, medication use, date, type of surgery, pre-operative status, intra-operative variables, post-operative course, tumor characteristics, complications, length of hospital stay, reinterventions, re-admissions and death. Readmission was defined as any additional hospitalization, excluding admission to subacute care or rehabilitation facility within 30 days [19,20]. All procedures were performed by experienced surgeons who had completed training in pancreatic and colorectal surgery. The general health condition of the patient was appraised using the Charlson comorbidity index [21]. All postoperative complications were assessed using the Clavien-Dindo classification system, a well-known and widely used system in the surgical field to uniformly classify and grade postoperative complications [22]. Data on the postoperative course were collected up to the 90th postoperative day.

The primary endpoint for this study was the 90-day Clavien-Dindo classification of surgical complications. Secondary endpoints were duration of surgery, intraoperative blood loss or iatrogenic injuries, infections and complications in wound healing, cardiovascular complications, organ failure, neurologic complications, gastro-intestinal complications, postoperative C-reactive protein, length of postoperative hospital stay, readmission within 30 days after discharge and death within 90 days after surgery. Pancreatic fistula, delayed gastric emptying (DGE) and postpancreatectomy hemorrhage were defined according to the International Study Group of Pancreatic Surgery definitions [23–25].

### Statistics

Statistical analysis was performed using the Statistical Package for the Social Science (SPSS, Chicago, IL), version 23. Continuous variables are presented as median values with interquartile ranges (IQRs) and categorical variables as frequencies, number of patients and percentages. The primary endpoint for this study, the 90-day Clavien-Dindo classification of surgical complications, was compared using the χ^2^–test. Other secondary outcomes were similarly compared between the two groups for categorical variables using the χ^2^–test or by the two-sided Fisher exact test as appropriate. For continuous variables, the student’s t-test and the nonparametric Mann-Whitney U test were used. A *P* value of 0.05 was considered statistically significant.

## Results

### Patient’s characteristics

In the jejunal IR group (n = 10), there were eight male and two female patients with a median age of 67 years (range 48–79 years). Eight (80%) patients had malignant disease and the remaining two (20%) patients had chronic pancreatitis.

To test whether the post-operative outcome or incidence and severity of complications were different in the experimental IR group, we analyzed a matched control group of 20 patients. When comparing both groups, no significant differences were found regarding comorbidities, tumor location, preoperative therapies, intoxications, or ASA classification. Furthermore, groups did not differ with respect to the frequency of positive resection margins. In both the experimental jejunal IR group and matched No IR group, positive margins (R1) were recorded in one patient. Patient’s characteristics, comorbidities and postoperative diagnoses are presented in Table 1.

**Table 1:** Demographics and comorbid conditions of patients undergoing pancreatic surgery with and without experimental in vivo jejunal IR.

N (%) or median (IQR)	All (30)	Jejunal IR (10)	No IR (20)	*P*
Sex				1.0*
• Male	24 (80)	8 (80)	16 (80)	
• Female	6 (20)	2 (20)	4 (20)	
Age (range)	67 (45–79)	67 (48–79)	65 (45–79)	0.83^†^
Charlson index score				1.0*
• 0–5	6 (20)	2 (20)	4 (20)	
• 5–10	24 (80)	8 (80)	16 (80)	
• >10	0 (0)	0 (0)	0(0)	
Tumor location				0.3*
• Pancreatic head	12 (40)	2 (20)	10 (50)	
• Papil of Vater	8 (26.7)	4 (40)	4 (20)	
• Extrahepatic biliary ducts	3 (10)	2 (20)	1 (5)	
• Duodenum	1 (3.3)	0 (0)	1 (5)	
• Benign / pancreatitis	6 (20)	2 (20)	4 (20)	
Coronary artery disease	4 (13.3)^a^	1 (10)	3 (15.8)^a^	1.0
Hypertension	10 (35.7)^a^	4 (40)	6 (33.3)^a^	1.0
Peripheral vascular disease	1 (3.4)^a^	1 (10)	0 (0)^a^	0.35
COPD	0 (0)^a^	0 (0)	0 (0)^a^	1.0
Diabetes mellitus	7 (24.1)^a^	2 (20)	5 (26.3)^a^	1.0
Preoperative pancreatitis	4 (14.8)^a^	0(0)	4 (23.5)^a^	0.26
Liver insufficiency	0 (0)^a^	0 (0)	0 (0)^a^	1.0
Preoperative jaundice	12 (85.7)^a^	3 (33.3)^a^	9 (52.9)^a^	0.43
Renal insufficiency	0 (0)^a^	0 (0)	0 (0)^a^	1.0
Previous abdominal surgery	9 (32.1)^a^	4 (44.4)^a^	5 (26.3)	0.4
Preoperative radiotherapy	0 (0)	0 (0)	0 (0)	1.0
Preoperative chemotherapy	0 (0)	0 (0)	0 (0)	1.0
Tobacco use^a^				1.0
• Ever	14 (66.7)	6 (66.7)	8 (66.7)	
• Never	7 (33.3)	3 (33.3)	4 (33.3)	
Alcohol use^a^				0.63*
• Current	14 (66.7)	7 (77.8)	7 (58.3)	
• Abusus	4 (19.0)	1 (11.1)	3 (25.0)	
• Never	3 (14.3)	1 (11.1)	2 (16.7)	
Body mass index (kg/m^2^)	23 (22–25)	25 (23–29)	22 (21–24)	0.19^†^
ASA^b^				0.41*
• 1	1 (3.4)	0 (0.0)	1 (5.3)	
• 2	22 (75.9)	9 (90.0)	13 (68.4)	
• 3	6 (20.7)	1 (10.0)	5 (26.3)	
• 4	(0.0)	0 (0.0)	0 (0.0)	
Surgical margin status				1.0
• R0	28 (93.3)	9 (90.0)	19 (95.0)	
• R1	2 (6.7)	1 (10.0)	1 (5.0)	

* χ^2^ test.

† Mann Whitney u test. Others: Fisher exact test. IQR = interquartile range.

COPD = Chronic obstructive pulmonary disease. ASA = American Society of Anesthesiologists. 1: Normal healthy patient. 2: Patient with mild systemic disease. 3: Patient with severe systemic disease that is not incapacitating. 4: Patient with severe, life-threatening systemic disease.

^a^ 4–9 missing values.

^b^ 1–3 missing values.

Eight male and two female patients with a median age of 60 years (range 45–66 years) were included in the colonic IR group (n = 10). To evaluate the safety of the experimental colonic IR protocol, these patients were compared with 20 control patients included in the same time period. All patients suffered from malignant disease. Low anterior resection (n = 26) was the most performed surgical technique compared with the abdominoperineal resection (n = 4). No significant differences were found regarding comorbidities, AJCC cancer stage, preoperative therapies, intoxications, or ASA classification of patients undergoing colorectal surgery with and without experimental in vivo colonic IR. All patients underwent an open colorectal procedure. No positive resection margins (R1) were recorded in either the experimental in vivo colonic IR group, nor in the matched No IR group. Patient characteristics, comorbidities and postoperative diagnoses are presented in Table 2.

**Table 2:** Patient demographics and comorbid conditions of patients undergoing colorectal surgery with and without experimental in vivo colonic IR.

N (%) or median (IQR)	All (30)	Colonic IR (10)	No IR (20)	*P*
Sex				1.0*
• Male	24 (80)	8 (80)	16 (80)	
• Female	6 (20)	2 (20)	4 (20)	
Age (range)	63 (45–79)	60 (45–66)	65 (48–79)	0.29^†^
Charlson index score				0.21*
• 0–5	9 (30)	4 (40)	5 (25)	
• 5–10	20 (67)	5 (50)	15 (75)	
• >10	1 (3)	1 (10)	0 (0)	
Tumor location				1.0*
• Pancreatic head	3 (10)	1 (10)	2 (10)	
• Papil of Vater	12 (40)	4 (40)	8 (40)	
• Extrahepatic biliary ducts	15 (50)	5 (50)	10 (50)	
• Duodenum	0 (0)	0 (0)	0 (0)	
• Benign / pancreatitis	3 (10)	1 (10)	2 (10)	1.0
Coronary artery disease	11 (37)	3 (30)	8 (40)	0.71
Hypertension	1 (3)	0 (0)	1 (5)	1.0
Peripheral vascular disease	3 (10)	0 (0)	3 (15)	0.53
COPD	6 (20)	2 (20)	4 (20)	1.0
Diabetes mellitus	4 (13)	1 (10)	3 (15)	1.0
Preoperative pancreatitis	1 (3)	0 (0)	1 (5)	1.0
Liver insufficiency	11 (37)	6 (60)	5 (25)	0.1
Preoperative jaundice	29 (100)	10 (100)	19 (100)	1.0
Renal insufficiency	25 (86)	8 (80)	17 (89)	0.6*
Previous abdominal surgery				1.0
Preoperative radiotherapy	12 (46.1)	3 (50.0)	9 (45.0)	
Preoperative chemotherapy	14 (53.8)	3 (50.0)	11 (55.0)	
Tobacco use^a^				0.8*
• Ever	12 (40)	3 (60)	9 (45)	
• Never	6 (20)	1 (20)	5 (25)	
Alcohol use	7 (23)	1 (20)	6 (30)	
• Current	27 (22–29)	27 (24–29)	26 (22–29)	0.66^†^
• Abusus				0.5*
• Never	8 (27)	3 (30)	5 (25)	
Body mass index (kg/m^2^)	18 (60)	7 (70)	11 (55)	
ASA	3 (10)	0 (0)	3 (15)	
• 1	1 (3)	0 (0)	1 (5)	
• 2				
• 3	30 (100)	10 (100)	20 (100)	*1*.*0*
• 4	0 (0)	0 (0)	0 (0)	
Surgical margin status	24 (80)	8 (80)	16 (80)	1.0*
• R0	6 (20)	2 (20)	4 (20)	
• R1	63 (45–79)	60 (45–66)	65 (48–79)	

* χ^2^ test.

† Mann Whitney u test. Others: Fisher exact test. IQR = interquartile range.

COPD = Chronic obstructive pulmonary disease. ASA = American Society of Anesthesiologists. 1: Normal healthy patient. 2: Patient with mild systemic disease. 3: Patient with severe systemic disease that is not incapacitating. 4: Patient with severe, life-threatening systemic disease.

^a^4-9 missing values.

### The incidence and severity of postoperative complications with or without in vivo jejunal IR

The overall postoperative complication rate using the Clavien-Dindo classification following pancreatoduodenectomy in the total population of 30 patients was 63.3%. 48 adverse events occurred in these 19 patients. The frequency and severity of postoperative complications were equal for patients undergoing pancreatic surgery with and without experimental in vivo jejunal IR. As shown in Table 3, grade 1 complications were not statistically different for both groups. Also, no significant differences were found for grade, grade 3a, grade 3b, grade 4a and 4b. Only one patient died (grade 5 complication) in the No IR group, the cause of death being sepsis with multiple organ failure following anastomotic leakage. No fatalities were recorded in the IR group.

**Table 3:** Morbidity and mortality of patients undergoing pancreatic surgery with and without experimental in vivo jejunal IR.

N (%) or median (IQR)	All (30)	Jejunal IR (10)	No IR (20)	P
*Primary endpoint*				
Patients with complications	19 (63.3)	7 (70)	12 (60)	0.7
Total complications	48	19	29	
Clavien Dindo score				0.23*
• 1	9 (30)	5 (50)	4 (20)	
• 2	13 (43.3)	4 (40)	9 (45)	
• 3a	13 (43.3)	5 (50)	8 (40)	
• 3b	6 (20)	2 (20)	4 (20)	
• 4a	4 (13.3)	3 (30)	1 (5)	
• 4b	2 (6.7)	0 (0)	2 (10)	
• 5	1 (3)	0 (0)	1 (5)	
*Secondary endpoints*				
Infection and wound healing				
Anastomotic leakage	9 (30)	3 (30)	6 (30)	1.0
• Intestinal necrosis	0 (0)	0 (0)	0 (0)	1.0*
• Pneumonia	3 (10)	3 (30)	0 (0)	0.10
• Wound infection	8 (26.7)	4 (40)	4 (20)	1.0
• Sepsis	6 (20)	2 (20)	4 (20)	1.0
• Wound dehiscence	0 (0)	0 (0)	0 (0)	1.0
• Intra-abdominal abscess	7 (23.3)	1 (10)	6 (30)	0.37
• Urinary tract infection	3 (10)	1 (10)	2 (10)	1.0
Cardiovascular complications				
• Central venous catheter infection	0 (0)	0 (0)	0 (0)	1.0
• Intra-abdominal hemorrhage	3 (10)	1 (10)	2 (10)	1.0
• Thromboembolic event	0 (0)	0 (0)	0 (0)	1.0
• Arrhythmia	1 (3)	0 (0)	1 (5)	1.0
• Myocardial infarction	0 (0)	0 (0)	0 (0)	1.0
• Cerebral vascular accident	1 (3)	1 (10)	0 (0)	1.0
Organ failure				
• Respiratory failure	1 (3)	0 (0)	1 (5)	1.0
• Hepatic failure	0 (0)	0 (0)	0 (0)	1.0
• Renal failure	1 (3)	0 (0)	1 (5)	1.0
Gastro-intestinal complications				
• Ileus	3 (10)	2 (20)	1 (5)	0.25
• DGE	5 (16.7)	4 (40)	1 (5)	0.03
• Fistulas	0 (0)	0 (0)	0 (0)	1.0
Postoperative CRP^a^	238 (96–452)	237 (103–450)	238 (96–450)	0.63^†^
Length of hospital stay	19 (10–31)	18 (9–38)	19 (11–29)	0.86^†^
Hospital readmission <30 days	5 (16.7)	2 (20)	3 (15)	0.58
Reoperation	6 (20)	2 (20)	4 (20)	1.0
Death < 90 days	1 (10)	0 (0)	1 (5)	1.0

* χ^2^ test.

† Mann Whitney u test. Others: Fisher exact test. IQR = interquartile range.

DGE indicates delayed gastric emptying. CRP = C-reactive protein.

^a^4 missing values.

In both the jejunal IR and No IR group anastomotic leakage, infection-related complications (wound infection and intra-abdominal abscesses), gastrointestinal and vascular adverse events represented the five most common postoperative complications. These complication rates were similar after jejunal IR and No IR, except for DGE. The incidence of DGE was significantly higher after jejunal IR compared to No IR. The median postoperative C-reactive protein value (CRP, postoperative day 3 +/- 1 day) and duration of hospitalization were not affected by inclusion of patients in the jejunal IR protocol.

### The incidence and severity of postoperative complications with or without in vivo colonic IR

Table 4 provides a summary of all postoperative complications observed in both colonic groups in terms of type and severity according to the Clavien-Dindo classification. Sixty percent of the patients experienced a complicated postoperative course after colorectal surgery. Overall 33 complications were found in these 18 patients. When comparing both groups, five patients (50%) in the colonic IR group and 13 patients (65%) in the No IR group had one or more postoperative complication. The difference in the incidence of complications was not statistically significant.

**Table 4:** Morbidity and mortality of patients undergoing colorectal surgery with and without experimental in vivo colonic IR.

N (%) or median IQR)	All (30)	Colonic IR (10)	No IR (20)	*P*
*Primary endpoint*				
Patients with complications	18 (60)	5 (50)	13 (65)	0.46
Total complications	33	7	26	
Clavien Dindo score				0.44*
• 1	9 (30)	3 (30)	6 (30)	
• 2	10 (33.3)	1 (10)	9 (30)	
• 3a	5 (16.7)	1 (10)	4 (20)	
• 3b	7 (23.3)	2 (20)	5 (25)	
• 4a	1 (3)	0 (0)	1 (5)	
• 4b	1 (0)	0 (0)	1 (5)	
• 5	0 (0)	0 (0)	0 (0)	
*Secondary endpoints*				
Infection and wound healing				
Anastomotic leakage	4 (13.3)	0 (0)	4 (20)	0.27
• Intestinal necrosis	1 (3)	0 (0)	1 (5)	1.0
• Pneumonia	3 (10)	0 (0)	3 (15)	0.53
• Wound infection	6 (20)	2 (20)	4 (20)	1.0
• Sepsis	1 (3)	0 (0)	1 (5)	1.0
• Wound dehiscence	2 (7)	1 (10)	1 (5)	1.0
• Intra-abdominal abscess	7 (23)	2 (20)	5 (25)	1.0
• Urinary tract infection	6 (20)	1 (10)	5 (25)	0.64
Cardiovascular complications				
• Central venous catheter infection	0 (0)	0 (0)	0 (0)	1.0
• Intra-abdominal hemorrhage	0 (0)	0 (0)	0 (0)	1.0
• Thromboembolic event	0 (0)	0 (0)	0 (0)	1.0
• Arrhythmia	0 (0)	0 (0)	0 (0)	1.0
• Myocardial infarction	0 (0)	0 (0)	0 (0)	1.0
• Cerebral vascular accident	0 (0)	0 (0)	0 (0)	1.0
Organ failure				
• Respiratory failure	1 (3)	0 (0)	1 (5)	1.0
• Hepatic failure	0 (0)	0 (0)	0 (0)	1.0
• Renal failure	0 (0)	0 (0)	0 (0)	1.0
Gastro-intestinal complications				
• Ileus	5 (13)	1 (10)	4 (20)	0.64
• DGE	0 (0)	0 (0)	0 (0)	1.0
• Fistulas	0 (0)	0 (0)	0 (0)	1.0
Postoperative CRP^a^	192 (56–425)	182 (80–221)	192 (56–425)	0.77^†^
Length of hospital stay	12 (9–16)	13 (8–16)	12 (9–15)	0.79^†^
Hospital readmission <30 days	4 (13)	2 (20)	2 (10)	0.59
Reoperation	4 (13)	0 (0)	4 (20)	0.27
Death < 90 days	0 (0)	0 (0)	0 (0)	1.0

* χ^2^ test.

† Mann Whitney u test. Others: Fisher exact test. IQR = interquartile range.

DGE indicates delayed gastric emptying. CRP = C-reactive protein.

^a^5 missing values.

Patients enrolled in the experimental colonic IR group showed no increase in complication rate in Clavien-Dindo grade 1, 2 and 3. Grade 4a and 4b complications were exclusively observed in the No IR patient cohort. No patients died (grade 5 complication) within a 90 days postoperative period in neither of the two groups.

When comparing the incidence of complications by diagnosis, the most common observed complications were anastomotic leakage, infection-related complications and gastrointestinal adverse events. Interestingly, although the rates of all complications were similar after colonic IR or No IR, anastomotic leakage only occurred in the No IR group but failed to reach statistical significance. The median postoperative C-reactive protein value (CRP, postoperative day 3 +/- 1 day) and hospitalization period were similar for both groups.

### Intraoperative complications with or without in vivo jejunal IR

Possible intraoperative complications associated with the experimental IR protocol were obtained from the patient’s health records and operative reports. Factors evaluated in the analyses included operative time, estimated blood loss and intraoperative hemorrhage or iatrogenic injuries.

As depicted in Table 5, the duration of surgery in patients subjected to the jejunal IR protocol was not significantly prolonged compared to the No IR group. Furthermore, the jejunal IR model did not lead to significantly more blood loss, intraoperative hemorrhage or iatrogenic injuries.

**Table 5:** Operative data of patients undergoing pancreatic surgery with and without experimental in vivo jejunal IR.

N (%) or median IQR)	All (30)	Jejunal IR (10)	No IR (20)	*P*
Duration of surgery (min)	317 (228–395)^a^	288 (239–380)^a^	353 (227–398)	0.49^†^
Vascular resection	4 (13)	1 (10)	3 (15)	1.0
Blood loss (mL)	550 (150–775) ^a^	600 (200–2000)^a^	500 (0–700) ^a^	0.83^†^
Intraoperative complications				
• Hemorrhage	2 (7)	0 (0)	2 (10)	0.54
• Iatrogenic injuries	0 (0)	0 (0)	0 (0)	1.0

* χ^2^ test.

† Mann Whitney u test. Others: Fisher exact test. IQR = interquartile range.

^a^1-3 missing values.

### Intraoperative complications with or without in vivo colonic IR

Table 6 gives a detailed overview of the surgical and perioperative outcomes in patients undergoing colorectal surgery with and without experimental colonic IR. There were no differences between the colonic IR group and No IR group regarding duration of surgery, total blood loss, intraoperative hemorrhage or iatrogenic injuries.

**Table 6:** Operative data of patients undergoing colorectal surgery with and without experimental in vivo colonic IR.

N (%) or median IQR)	All (30)	Colonic IR (10)	No IR (20)	*P*
Duration of surgery (min)	251 (199–314)^a^	274 (251–329)^a^	228 (182–306)^a^	0.08^†^
Vascular resection	0 (0)	0 (0)	0 (0)	1.0
Blood loss (mL)	600 (100–1758)^a^	500 (0–2049) ^a^	600 (100–1758) ^a^	0.77^†^
Intraoperative complications				
• Hemorrhage	5 (17)	2 (20)	3 (15)	1.0
• Iatrogenic injuries	1 (3)	1 (10)	1 (5)	1.0

* χ^2^ test.

† Mann Whitney u test. Others: Fisher exact test. IQR = interquartile range.

^a^1-3 missing values.

## Discussion

In this study we describe the safety of two human experimental models to study IR of the small intestine and colon. Intestinal ischemia is a life threatening, frequently observed event associated with persistently high mortality and morbidity rates [3]. To improve patient outcome it is important to provide better insight in the pathophysiological mechanisms of human intestinal IR and to evaluate potential modalities for the early diagnosis of intestinal ischemia [1,4,9].

Using both models we were able to elucidate several key processes involved in the pathophysiology of human intestinal IR [14,26–31]. During the inclusion period no serious adverse events were reported in our patients undergoing the experimental IR protocol. However, no structural evaluation of complications following our human in vivo intestinal IR was yet provided, even though adverse events were accurately tracked and reported back to the medical ethical committee. Moreover, we wanted to evaluate whether patients with IR were equal compared with No IR patients regarding perioperative complication rates. As 60 minutes of ischemia is associated with the most morphological/histological intestinal damage and increased systemic inflammation, patients subjected to these ischemic time periods were likely to have the biggest risk of complications [15,16]. Therefore we focused on this group for our safety and complication analysis.

First our cohorts of patient with jejunal or colonic in vivo intestinal IR were well matched with control patients without in vivo IR, with respect to age, sex, tumor characteristics/classification, operation period and Charlson comorbidity index.

We observed a similar percentage of complications after jejunal in vivo IR compared to surgery alone (No IR) regarding all complications as classified by the Clavien-Dindo system. Pancreatoduodenectomies are complex abdominal operations with high risk of morbidity and mortality. In our study, this rate is 63.3%, which is in agreement with the current literature [32,33]. When analyzing the complications by diagnosis, a significantly higher incidence of 40% of DGE was found in the jejunal IR group. Such rates of DGE after pancreatoduodenectomies, however, have also been reported by Glowka et al. with incidences as high as 61% [34]. Mortality was 5% in our total population (IR groups and matched controls). This is in concordance with mortality rates reported in previous studies revealing that high-volume centers significantly improve survival rates [35]. Operative time and blood loss were not different between our patient groups and previously reported data [36,37].

The overall complications rates in our patients undergoing colorectal surgery with and without experimental in vivo colonic IR did not differ significantly between the IR and No IR group, but are higher than mentioned in recently published reports, demonstrating an overall complication rate of 20% up to 51% [38–40]. A potential reason for this discrepancy seems to be the meticulous documentation of all complications according to the Clavien-Dindo classification system in our analysis. In both groups most patients with a complicated postoperative course experienced only minor complications. The incidence of anastomotic leakage in our cohort is 13.3% (4/30 patients), which is similar in comparison to recent literature reporting rates of 13.4% up to 20.0% after a follow-up of more than 30 days [41]. The recorded amount of blood loss, other intraoperative complications and length of hospital stay parallels results of the open surgery groups from the recent COLOR and CLASICC trails [38,39].

Next to that, the positive surgical margin rates in both the in vivo jejunal IR group and colonic IR group did not differ when compared to their respective matched control groups, reflecting oncologically adequate and safely executed surgical procedures, despite being subjected to the experimental IR-model. Finally, no significant iatrogenic injuries were observed in the IR groups, even though extra intraoperative manipulation of the intestine and vascular supply was present and extra surgical tools were used. These favorable intraoperative data add substantially to the argument that it is safe to study in vivo human intestinal IR by applying intestinal ischemia to six cm of isolated jejunum or colon during surgery, according to the described protocols.

Our study has several limitations. Because of the retrospective study design, some (minor) postoperative complications might have been missed. Furthermore, comparability with other studies using the same Clavien-Dindo grading system for severity of postoperative complications could be limited since inter-observer reproducibility of the classification system might vary. However, this study was not designed to test whether our postoperative complication rates were superior or inferior to previously reported complication rates in the literature. Some relatively infrequent occurring complications might not have been observed and recorded because of their naturally low incidence and might therefore not show a significant difference between the IR groups and their matched controls. Furthermore, because of the relatively low sample number, sampling errors might have occurred, rendering extrapolation of a comprehensive safety profile more difficult.

By using the human experimental models described in this study, unravelling of histopathological, functional and molecular sequelae that take place during human jejunal or colonic intestinal IR has been made possible and no translation or extrapolation from animal studies is necessary. Since we cannot totally prevent intestinal IR, future research should be directed at identifying potential therapeutic targets in the treatment of human intestinal IR-induced injury. By using the human IR models, different compartments of the intestine, for example the epithelial compartment, can be studied in more detail in the search for means to ameliorate intestinal IR-induced injury and improve patient outcome and survival.
